# Probing a southern hemisphere VLBI Intensive baseline configuration for UT1 determination

**DOI:** 10.1186/s40623-022-01671-w

**Published:** 2022-07-28

**Authors:** Sigrid Böhm, Johannes Böhm, Jakob Gruber, Lisa Kern, Jamie McCallum, Lucia McCallum, Tiege McCarthy, Jonathan Quick, Matthias Schartner

**Affiliations:** 1 Department of Geodesy and Geoinformation, TU Wien, Wiedner Hauptstraße 8-10, 1040 Vienna, Austria; 2 University of Tasmania, Hobart, Australia; 3 Hartebeesthoek Radio Astronomy Observatory, Krugersdorp , South Africa; 4 ETH Zürich, Zurich, Switzerland

**Keywords:** Very Long Baseline Interferometry (VLBI), UT1−UTC, Intensive sessions, Earth rotation, VLBI mixed-mode

## Abstract

The deviation of Universal Time from atomic time, expressed as UT1−UTC, reflects the irregularities of the Earth rotation speed and is key to precise geodetic applications which depend on the transformation between celestial and terrestrial reference frames. A rapidly varying quantity such as UT1−UTC demands observation scenarios enabling fast delivery of good results. These criteria are currently met only by the Very Long Baseline Interferometry (VLBI) Intensive sessions. Due to stringent requirements of a fast UT1−UTC turnaround, the observations are limited to a few baselines and a duration of one hour. Hence, the estimation of UT1−UTC from Intensives is liable to constraints and prone to errors introduced by inaccurate a priori information. One aspect in this context is that the regularly operated Intensive VLBI sessions organised by the International VLBI Service for Geodesy and Astrometry solely use stations in the northern hemisphere. Any potential systematic errors due to this northern hemisphere dominated geometry are so far unknown. Besides the general need for stimulating global geodetic measurements with southern observatories, this served as a powerful motivation to launch the SI (Southern Intensive) program in 2020. The SI sessions are observed using three VLBI antennas in the southern hemisphere: Ht (South Africa), Hb (Tasmania) and Yg (Western Australia). On the basis of UT1−UTC results from 53 sessions observed throughout 2020 and 2021, we demonstrate the competitiveness of the SI with routinely operated Intensive sessions in terms of operations and UT1−UTC accuracy. The UT1−UTC values of the SI reach an average agreement of 32 µs in terms of weighted standard deviation when compared with the conventional Intensives results of five independent analysis centers and of 27 µs compared with the 14C04 series. The mean scatter of all solutions of the considered northern hemisphere Intensives with respect to C04 is at a comparable level of 29 µs. The quality of the results is only slightly degraded if just the baseline HtHb is evaluated. In combination with the e-transfer capabilities from Ht to Hb, this facilitates continuation of the SI by ensuring rapid service UT1−UTC provision.

**Graphical Abstract:**

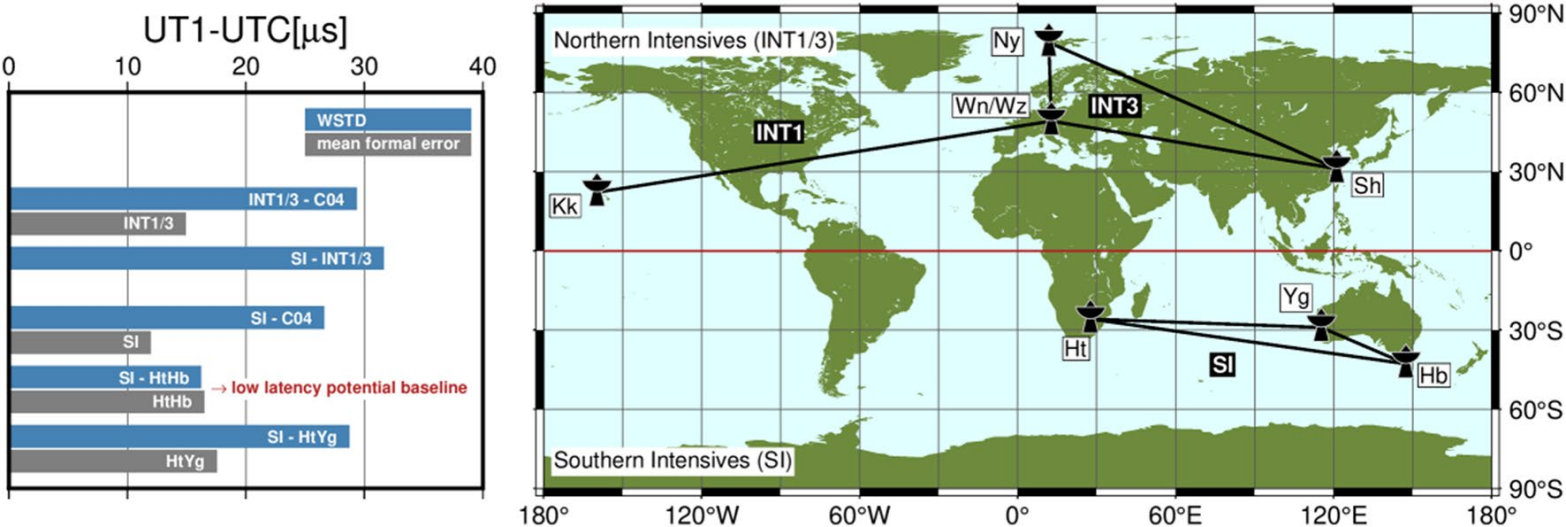

## Introduction

The quantity UT1−UTC is the difference between UT1 (Universal Time 1), which can be taken as the time determined by the rotation of the Earth (IERS Conventions 2010), and the uniform UTC (Coordinated Universal Time) based on atomic clocks. Representing one of the five Earth orientation parameters (EOP), UT1−UTC is essential for the conventional transformation between the terrestrial and the (geocentric) celestial reference systems. The other four parameters are the coordinates of the celestial intermediate pole in the terrestrial reference frame, describing polar motion, and its angular position w.r.t. the celestial reference frame, i.e. precession/nutation. The EOP are determined from measurements of the space geodetic techniques and provided by the International Earth Rotation and Reference Systems Service (IERS). Due to the correlation of the orbital elements of the satellites with UT1−UTC, as well as with nutation offsets, satellite-based techniques are not suited for the direct estimation of these parameters, but enable the estimation of the respective rates, as explained by Rothacher et al. (1999).

Geodetic Very Long Baseline Interferometry (VLBI) determines the International Celestial Reference Frame (ICRF, Charlot et al. 2020) and contributes to the International Terrestrial Reference Frame (ITRF, Altamimi et al. 2016) with essential information, e.g. the global scale. Furthermore, and specifically relevant in the context of this work, VLBI is currently the only technique capable of measuring the full set of EOP. Global observations are organised through the international VLBI Service for Geodesy and Astrometry (IVS, Nothnagel et al. 2017), with regular observing sessions of 24-h duration performed at least twice a week. The turnaround times between the actual observations and geodetic results are typically about 15 days for the fastest sessions, but can reach up to several months for others. Such long latencies are particularly unacceptable for a highly variable parameter such as UT1−UTC. In order to provide both rapid service UT1−UTC results and fill the observational gaps between the 24-h sessions, the so-called Intensive sessions or Intensives (INT) were established. The INT are typically single or triple baseline sessions with a duration of one hour. These experiments are performed every day and their limited nature allows for a moderately fast turnaround time, with results, at best, available within a few hours after the observation. We estimate the current median latency of standard INT sessions to be around one day.

One disadvantage of the current INT series is that there is little room for redundancy or control. One session usually has between 15 and 40 observations per baseline and, paired with the limited geometry of one baseline (or multiple in some cases), this only allows for a limited number of parameters to be estimated. The limitation in the analysis subsequently implies that the derived UT1−UTC results are sensitive to errors in the chosen a priori values for the fixed parameters, such as station coordinates or polar motion and nutation offsets. This sensitivity has been clearly shown by Nothnagel and Schnell (2008) and is currently investigated in more detail by Kern et al. (submitted to *J Geodesy*).

**Fig. 1:**
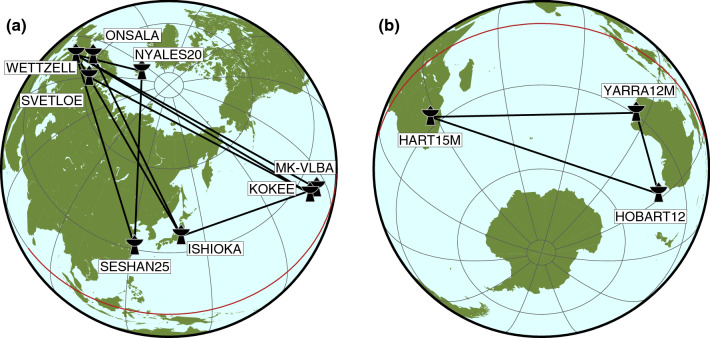
Stations and baselines of INT sessions. **a** Northern hemisphere configurations typically involved in IVS INT sessions. **b** Network of the SI program

The long-serving Intensive series are INT1, operated Monday to Friday on a baseline extending from Hawaii (Kokee, sometimes MK-VLBA) to Wettzell in Germany (sometimes also accompanied by Svetloe in Russia), and INT2, completing the daily series on the weekends with observations on the baseline Wettzell to Tsukuba (later Ishioka) in Japan. Since 2007 the INT1 and INT2 are supplemented with the INT3 sessions, observed on Mondays mostly on a triangle configuration, which includes Ny-Ålesund on Spitsbergen (Norway) in addition to Wettzell and the Japanese station (sometimes also Seshan25 in China). Figure 1a shows a map of the VLBI sites which routinely, or at least frequently, take part in the IVS Intensive sessions.

With the commissioning of more and more telescopes of the next-generation VLBI system, VGOS (VLBI Global Observing System; Niell et al. 2018), a new line of VGOS Intensives was started in 2020. Haas et al. (2021) went ahead with the VGOS-B sessions, which involve observations using the Onsala twin telescopes in Sweden and again the station Ishioka, which is capable of switching between legacy S/X and VGOS mode. Other VGOS Intensives are now observed nearly every weekday, employing the modern United States antennas in North America and on the Hawaiian islands, and one of the Wettzell twin telescopes. These developments add more redundancy, as UT1−UTC can be estimated from different measurements, which are largely independent from each other. Yet, when looking at the VLBI sites participating in Intensive sessions, we quickly realise that none of the series include a station located south of the equator. Since parameters like station positions and polar motion or nutation offsets cannot be estimated within the analysis of an Intensive session, such a lopsidedness bears the risk of introducing systematic effects, which are not compensatable or even detectable with observations stemming from solely northern networks. In a study, which is finalised in parallel to this article, Kern et al. (submitted to *J Geodesy*) corroborate by means of simulations that the effect of, for example, polar motion errors on UT1−UTC estimated from a northern baseline is opposite to the impact on UT1−UTC derived from a baseline in the South. Of course, this imbalance is not an Intensive-specific problem, the southern hemisphere has traditionally been under-represented in global VLBI measurements, an effect also visible in the end results (cf. Titov 2007).

In the early 2000s, the AuScope VLBI array (Lovell et al. 2013) was built to address this imbalance, with three 12-m telescopes on the Australian continent, one each in Hobart, Katherine and Yarragadee. Soon amongst the busiest IVS telescopes, the AuScope VLBI array had an impact on global results. Plank et al. (2015) reported that the precision of southern baselines finally caught up with that of northern ones. Unfortunately, for the next-generation VLBI system, VGOS, the southern hemisphere again seems to be falling behind, with the current VGOS network consisting of solely northern telescopes (Niell et al. 2018).

Given the unbalanced geographic distribution of stations participating in INT sessions combined with the emerging infrastructure for high-precision geodetic VLBI in the global South, we initiated the Southern Intensives (SI) program in late 2019. The main objective of this program is to establish an Intensive baseline independent of the “usual” INT networks, in order to get additional UT1−UTC estimations that are effective for rigorous verification. This article is primarily considered a proof of concept of the SI program, while the final objective, to equalise the current geographical bias, should be achieved with regularly observed SI sessions in the future. The Southern Intensive baselines are illustrated in Fig. 1b. According to Schartner et al. (2021a), the selected southern network of Hartebeesthoek (HART15M, Ht), Yarragadee (YARRA12M, Yg) and Hobart (HOBART12, Hb) fits a preferred geometry and should deliver good UT1−UTC results.

Besides generating additional UT1−UTC estimates, a dedicated goal of the SI is to promote the use of southern telescopes and boost their performance and expertise in cadence, production results, and session management. As demonstrated in the AUSTRAL campaign (Plank et al. 2017), active involvement in the full process (i.e., session planning and scheduling, correlation, post-processing and final analysis) proved beneficial for rapid improvement and innovation and was essential in gaining a comprehensive understanding of the VLBI technique. In addition, more regular sessions trigger improvements in data transport and processing chains, an area still requiring significant development globally in order to match VGOS expectations.

It should also be mentioned here that Hb has been upgraded to a VGOS system with work underway to achieve full compatibility with the current IVS VGOS program. While not active in standard legacy VLBI observations at the moment, Hb is regularly participating in so-called mixed-mode observations (Niell et al. 2021; McCallum and McCallum 2019). The AUSTRAL series and a dedicated Australian mixed-mode series adopted a legacy mode allowing the new VGOS system in Hobart (and Katherine) to participate (McCallum et al. submitted to *J Geodesy*), and this mode is also used for the Southern Intensives. In the study mentioned before as well as in this work, it can be shown that the analysis results obtained from the mixed-mode observations are of comparable quality as the results from standard legacy S/X mode observations.

An essential aspect for the determination of UT1−UTC with VLBI is the calibration of the UTC time-tags, which are assigned to the VLBI raw data by the station’s digital backends. For the SI sessions, the calibration method based on the concept of peculiar offsets (Himwich et al. 2017; described in more details in section “Observation and correlation”) is chosen, which is also used for the other sessions within the IVS, including Intensives. This ensures that the UT1−UTC values of SI can be compared to the results of IVS INT sessions without further correction. Since this calibration method is directly related to the instrumental delay of a station, a peculiar offset was calculated for the new receiving system of Hobart in this study to achieve highly accurate UT1−UTC values from SI.

In this paper, we present the setup, procedures and results of 53 SI sessions observed from January 2020 to December 2021. In section “Scheduling and simulations”, we briefly describe the scheduling strategy, which is further backed by simulation results. Section “Observation and correlation” contains the specifications of data recording and transfer as well as a dedicated block about the determination of the so-called peculiar offset for Hb as part of the correlation process. The estimation of UT1−UTC using the VLBI analysis module of VieVS (Böhm et al. 2018, Vienna VLBI and Satellite Software) is explained in section “Data analysis and results”. Within that section, we also assess the performance of the SI in comparison with other Intensive sessions, applying the continuous UT1−UTC time series of the IERS EOP 14C04 solution from Bizouard et al. (2019) as a kind of benchmark. In preparation for the planning of future SI sessions, in the last part of the results section we investigate the quality of the SI intrinsic single baselines HtHb and HtYg as compared to the triple baseline configuration HtHbYg. Finally, we give a wrap-up discussion and an outlook for the SI program in section “Conclusions and future perspectives”.

## Scheduling and simulation

The SI observed in 2020 were scheduled on Monday, Tuesday or Wednesday, either at 18:30 UTC, simultaneous with an INT1 session, or at 15:30 UTC, usually between an INT3 and INT1 session. Due to practical considerations, we shifted the observation plan for 2021 to Fridays at 18:45 UTC, again in parallel to INT1 sessions.

The schedules are generated using VieSched++ (Schartner and Böhm 2019) using a dedicated Intensive scheduling algorithm, presented in Schartner et al. (2021a, Appendix A). This algorithm tries to put special focus on observing sources located at the cusps of the mutually visible sky (Nothnagel and Campbell 1991), since previous work by Uunila et al. (2012) and Gipson and Baver (2015) proved that these observations have the biggest impact on achieving high UT1−UTC accuracy. Specifically, as done in Haas et al. (2021) for VGOS Intensives between Ishioka and Onsala, the algorithm tries to observe such sources every 10 min.

In between these observations, a standard geodetic scheduling approach is used that optimises the station sky-coverage while trying to ensure a high number of scans. Since it is not possible to fulfil both requirements at the same time, as discussed in Gipson (2010) and proven by Schartner and Böhm (2020), it is necessary to find a good balance between them. To optimise the remaining observations, a brute-force approach is used, testing various different optimisation criteria weights resulting in the generation of a multitude of different schedules per session. This brute-force approach was guided by an iterative evolutionary strategy for scheduling parameter optimisation as discussed in Schartner et al. (2021b). Every schedule is further simulated and analysed one thousand times to determine the theoretical UT1−UTC mean formal error and repeatability value. The simulated error contributions consist of three parts (Pany et al. 2011): tropospheric turbulences considering spatial and temporal correlation, clock variations, and white noise. Based on these simulation results, the best schedule is selected and distributed to the stations.

Ultimately, this scheduling strategy leads to between 30 and 40 scans for the three station network resulting in 90 to 120 observations. Figure 4 in section “Data analysis and results” displays the number of observations scheduled (normalised by the number of baselines) and the mean formal errors resulting from the simulations, together with the normalised number of observations used in the analysis of the SI sessions, as well as the corresponding UT1−UTC formal errors.

## Observation and correlation

The Southern Intensives are observed in the AUSTRAL mode, using 10 channels in X-band and 6 channels in S-band with a width of 16 MHz and by using 2-bit sampling. This gives a total data rate of 1 Gbps. Due to prominent radio frequency interference in Hobart, the only common spectrum left in S-band is $\sim$ 100 MHz between 2.2 and 2.3 GHz. As such, we adopt a contiguous channel configuration between 2.201 and 2.297 GHz.

As mentioned above, Hb has been upgraded to VGOS and joins these observations with the new receiver and backends. Compatibility with the legacy mode is ensured by preserving signals below 3 GHz via the old coaxial cable connection, while frequencies above are sent on the fibre-optic cable (RFoF) to the control room. In order to match the right-circularly polarised signal from the legacy systems, in Hobart both linear polarisations (X, Y) are used in the correlation stage and combined in the fringe fitting stage using Fourfit (part of the HOPS package MIT/Haystack 2021). Besides the second polarisation, current DBBC3 modes only allow for 32 MHz channels, leading to a 3 Gbps data stream that needs to be recorded at Hobart. A detailed description of the mixed-mode observations applied in the SI sessions is given in McCallum et al. (submitted to *J Geodesy*).

Each session has about 30 scans per station, with an average on source time of 60 s. This means a total data volume of about 250 GB per station. A good data connection to HartRAO (Hartebeesthoek Radio Astronomy Observatory) allows the data to be e-transferred to Hobart, typically within a couple of hours. Unfortunately, from Yarragadee the data need to be physically shipped, with a typical delay of 5–7 days.

The time tags of the recorded data produced by the digital backend of a VLBI station are used to calculate the observation epoch for geodetic analysis. Since most of the geodetic analysis packages require the observation epoch to correspond to the epoch when the incoming wavefront from the radio source passes the antenna reference point, the time tags must be corrected for the delay between the reference point and the sampler and for the delay of the UTC reference signal between the GPS (Global Positioning System) receivers and the sampler (herein referred to as station delay). Neglecting the station delay impacts the UT1−UTC estimation, as a time tag shift translates into a UT1 shift of the same magnitude and opposite sign (Clark 1997). In order to provide UT1−UTC estimates from SI sessions that are consistent with the products of other IVS VLBI sessions, the same procedure to correct the station delay at the raw data level is used. This procedure also ensures consistency of UT1−UTC estimates between SI sessions.

Measuring the above-mentioned station delays is difficult. Instead it is common practice to determine this delay empirically during correlation. Within the IVS, the so-called peculiar offset is used and applied in the clock modelling at the correlator to shift the time tags of the recorded data to correct for the station delay. The peculiar offset is available for each station within the IVS and maintained by the United States Naval Observatory (USNO).^1^ The origin of the peculiar offset dates back to 1990 when the station delay was set to zero for Kokee. Since the Kokee station delay was not truly zero, the peculiar offsets of the other VLBI stations represent the difference between the station delay and the Kokee station delay. Furthermore, the UT1−UTC estimates provided by VLBI are biased by the station delay of Kokee from 1990 compared to true UT1−UTC values (Corey and Himwich 2018). The concept of peculiar offsets is also used for correlator clock modelling for the SI sessions. Hence, the UT1−UTC estimates from SI are also biased by this offset, but they are consistent with the UT1−UTC estimates provided by other IVS experiments.

In agreement with other IVS sessions, the clock offset of the correlator clock model is calculated for each SI session as (Himwich et al. 2017):

1 $$\begin{aligned} \tau _{\mathrm {clk},i}=\tau _{\mathrm {fmout-gps},i}+\tau _{\mathrm {pec},i}, \end{aligned}$$

where $\tau _{\mathrm {fmout-gps},i}$ is measured by each station *i* and represents the delay between the time tag assigned to the data (fmout) and UTC (GPS). In addition, a rate is estimated from the $\tau _{\mathrm {fmout-gps},i}$ measurements within each session and applied in the correlator clock model. For the SI station network consisting of Ht, Yg, and Hb, the peculiar offset $\tau _{\mathrm {pec}}$ is available for the stations Ht ($\tau _{\mathrm {pec,Ht}}=2.943$ µs) and Yg ($\tau _{\mathrm {pec,Yg}}=2.278$ µs). The peculiar offset for Hb is unknown since it has been equipped with a new broadband receiving system which changes the station delay. Hence, Eq. (1) cannot be applied directly for Hb. In order to align the raw data stream of Hb with Ht and Yg, a pre-correlation pass with an increased delay search window with DiFX-2.6.2 (Deller et al. 2011) is carried out by setting $\tau _{\mathrm {pec}}$ for Hb to zero. The resulting single band delay ($\tau _{\mathrm {SBD,Hb-Yg}}$) value obtained by fourfit (Cappallo 2017) on a baseline with Yg as reference station is used to correct the preliminary clock offset of Hb. The Hb clock offset for correlation is calculated as:

2 $$\begin{aligned} \tau _{\mathrm {clk,Hb}}=\tau _{\mathrm {fmout-gps,Hb}}+\tau _{\mathrm {SBD,Hb-Yg}}. \end{aligned}$$

This procedure ensures that the clock offset for Hb is consistent with the clock offsets for Ht and Yg, because it is tied to the clock offsets including the peculiar offset from the other stations. The corrected initial clock offset is then used in the main pass for correlation and fringe-fitting, yielding group delay reference epochs to estimate UT1−UTC values, which are consistent with other IVS experiments. Since the $\tau _{\mathrm {SBD}}$ represents a measurement of the peculiar offset of Hb (as can be seen by comparing (1) and (2)), the $\tau _{\mathrm {SBD,Hb-Yg}}$ values of seven SI sessions in 2020 are used to estimate the peculiar offset for Hb. For each SI session the clock offsets are calculated using (1) and (2). The average value of the resulting $\tau _{\mathrm {SBD,Hb-Yg}}$ is then used as an estimate for the Hb peculiar offset:

3 $$\begin{aligned} \tau _{\mathrm {pec,Hb}}=\frac{1}{7}\sum _{i=1}^{7}\tau _{\mathrm {SBD,Hb-Yg},i}. \end{aligned}$$

**Fig. 2:**
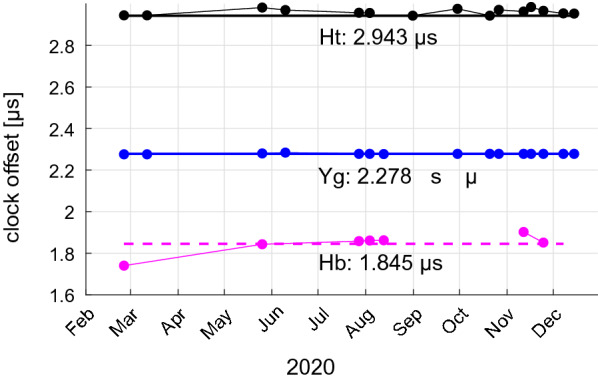
Correlation clock offsets corrected by $\tau _{\mathrm {fmout-gps}}$ measurements displayed as dots per SI session in 2020 for Ht (black), Yg (blue), and Hb (magenta). The solid lines represent the known IVS peculiar offsets for Ht and Yg, whereas the dashed line represents the estimated peculiar offset for Hb based on SI data

Equation (3) yields a peculiar offset of 1.845 µs for Hb with a root mean square value of 50 ns. This estimated peculiar offset for Hb is then used in the correlation clock model for the SI sessions in 2021, which makes it possible to carry out the main pass immediately and avoid the pre-correlation pass. This also helps to achieve shorter turnaround times which are critical for Intensive sessions. Since the discrepancy of 50 ns between the time tags of the raw data streams is significantly lower than the precision of UT1−UTC estimates, which is around 12 µs as discussed in section “Data analysis and results”, we expect consistent UT1−UTC estimates between SI sessions. The correlation clock offsets used for the SI sessions corrected by $\tau _{\mathrm {fmout-gps}}$ agree very well with the standard set of peculiar offsets which warrants consistency with UT1−UTC estimates from other IVS VLBI sessions (see Fig. 2).

The clock model for Ht is corrected with respect to the reference station Yg by small offsets in the order of several to tens of nanoseconds. These corrections are carried out to bring $\tau _{\mathrm {SBD,Ht-Yg}}$ close to zero which maximises the signal-to-noise ratio of the correlation product.

## Data analysis and results

The long-term objective of the Southern Intensives program is to provide additional UT1−UTC values, which are independent of the traditional Intensive networks, but of a comparable standard. This study aims at demonstrating the feasibility and assessing the currently attainable precision. Our approach to measure the quality of the SI UT1−UTC estimates is to compare them with the results of other Intensive sessions, as well as with the continuous UT1−UTC time series of the IERS 14C04 (C04, Bizouard et al. 2019). Regarding the comparison with the C04, we certainly have to be aware that the UT1−UTC series is calculated from a combination of different IVS combined and individual analysis center (AC) products. Which session types are introduced from which analysis centers and which weights they are given is unfortunately not transparent for a special period of interest. So we can only speculate that the C04 UT1−UTC are also influenced by results of Intensive sessions and the solutions used for the C04 could as well overlap with AC solutions selected for this study. Accordingly, we do not regard the C04 as a completely independent kind of ground truth, but rather as another comparative series that incorporates the results of the more precise 24-h sessions as well. A direct comparison with the UT1−UTC results of IVS R1 and R4 sessions is not implemented, because not all of the SI sessions are sufficiently close to an R1 or R4 session to keep the extrapolation error tolerably small.

The other Intensive session types are the IVS INT1 and INT3 sessions analysed by different IVS analysis centers and the so-called Russian Intensives (RI). Apart from INT1 and INT3 the RI are the only other Intensive baseline configuration with observation epochs reasonably close to the SI epochs. The RI are observed daily on one baseline of the Quasar network (Finkelstein et al. 2011), which consists of the three radio astronomical observatories Svetloe, Badary and Zelenchukskaya. The UT1−UTC results and uncertainties of the IVS Intensives and the databases of the RI are publicly available (see section “Availability of data and materials”). The SI results, the INT1/3 results and the RI results of the AC at TU Wien (vie) are determined by means of the VieVS VLBI module (links to the vie results and solution description are also provided in section “Availability of data and materials”).

### Processing of Intensives with VieVS

In the frame of this article, we analysed data files of 53 SI, 64 INT1/3 and 106 RI sessions using the same processing settings. For the a priori modelling of troposphere delays and site displacement, we apply standard geophysical models as recommended by the IERS and IVS. The specific choices, including details about the processing scheme set in VieVS, are documented in the solution description. The a priori EOP lines are calculated from the so-called “finals” (essentially the values belonging to IERS Bulletin A) using Lagrange interpolation. Whenever UT1−UTC is interpolated in the course of this work, the model for zonal tidal variations as recommended in the IERS Conventions (2010) is removed before and reapplied after interpolation, regardless of the adopted interpolation method. We did not use the C04 EOP series in the session analysis, because in a realistic Intensive timeline the sessions are analysed shortly after the observation, when C04 values are not yet available. High-frequency ocean tidal variations in polar motion and UT1 are taken into account with the model presented in Desai and Sibois (2016), which is also used by all space geodetic techniques for their ITRF2020 contributions. Station coordinates are fixed to the VieVS internal vievsTrf, since the ITRF2014 positions (Altamimi et al. 2016) of the Australian stations are merely based on about three years of data, as raised by McCallum et al. (submitted to *J Geodesy*). In the VieVS version that was used for processing, the vievsTrf positions and velocities correspond to the ones published online as vie2020_211030_withVGOS^2^. This TRF was calculated in a VLBI global solution, based on the sessions submitted to the IVS by AC vie, as part of the IVS contribution to the upcoming ITRF2020. Source positions are set to the a priori values given in the ICRF3 catalogue (Charlot et al. 2020). As it is standard for the analysis of Intensive sessions, the only parameters estimated are one linear clock function with respect to the reference clock per station, one zenith wet delay per station and one UT1−UTC offset. The reference epoch for UT1−UTC is the effective middle of the session, calculated from the times of the first and last scan.

**Fig. 3:**
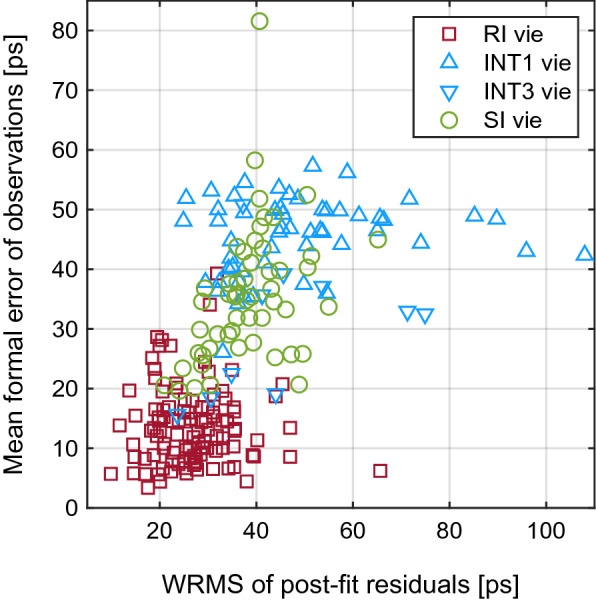
WRMS (weighted root mean square) of post-fit residuals [ps] from VieVS analysis versus mean formal error of observations [ps] from original databases

As it is standard in VieVS, the solutions are weighted using the formal errors of the observations with a constant noise of 0.5 cm (17 ps) added. The constant noise is added for a more realistic analysis, because it is commonly assumed that the uncertainties of the observations after fringe fitting are too optimistic. The mean formal errors of the observations per session, taken from the original databases, are displayed in Fig. 3 together with the WRMS (weighted root mean square) of the post-fit residuals for all three analysed session types (SI, INT1/3, RI). The mean and median values of the formal errors computed from all observations of the analysed sessions are listed in Table 1, labelled as $\sigma _{\mathrm {obs}}$. Figure 3 as well as the entries in the table show that, in spite of different observing modes, the uncertainties of the SI observations are of a similar size as those of the INT1/3 observations. The mean observation uncertainties and also the WRMS of the post-fit residuals of the RI sessions are remarkably smaller. These discrepancies are probably due to differences in the correlation and post-correlation procedure, where the formal errors of the delays result from.

### Scheduling and simulation versus analysis

**Fig. 4:**
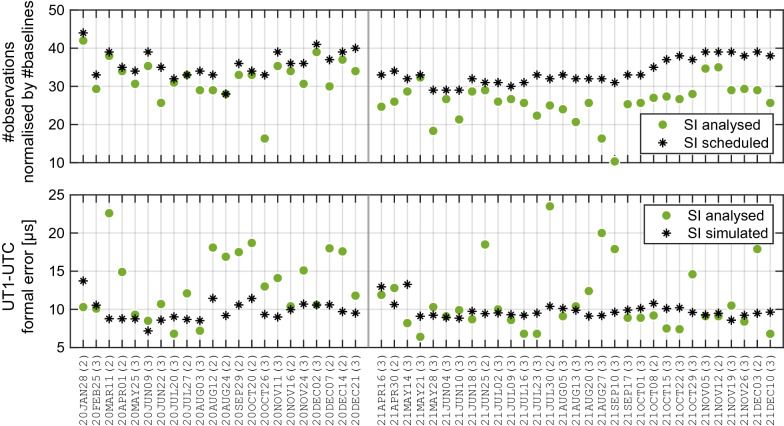
Top: number of observations normalised by number of baselines of the SI sessions as scheduled (black stars) and as used in the analysis (green circles). Bottom: UT1−UTC (mean) formal error in µs of the SI sessions as simulated (black stars) and as used in the analysis (green circles). The labels on the x-axis show the dates of the SI sessions and in braces the number of stations used in the analysis

In this section, we briefly investigate the agreement of scheduled and simulated SI with the analysed SI observations. The top panel of Fig. 4 shows the number of observations normalised by the number of baselines for the SI as scheduled and as used in the analysis for each session. We notice that about 18 % of the SI observations per baseline get lost in the process from scheduling to analysis, with this loss being more distinct in 2021. The explanation for this significant data loss is partly a too optimistic assumption about the antenna sensitivity for the new system in Hobart and even more importantly, significant performance issues with the new sampler in Hobart. After a repair and upgrade, as well as adjustment in the antenna sensitivity, more recent SI sessions do not show such a significant loss of observations.

The lower panel of Fig. 4 compares the UT1−UTC formal errors from the analysis with the mean formal errors resulting from the SI simulations. The numbers in braces after the SI dates denote if the sessions were finally observed or analysed with two or three stations. In the majority of the cases the formal errors of the analysis results are within $\pm\,2$ µs distance from the simulated mean formal errors or slightly smaller. Most of the sessions where we see larger formal errors of the analysis results are processed with two stations only. Comparing the means or medians of the SI simulated UT1−UTC mean formal errors and of the UT1−UTC formal errors from SI analysis, which are presented in Table 1 (labelled as $\sigma _{\mathrm {UT1}}$), we can state that the precision of the real observations meets the expectations very well.

### Validation against UT1–UTC results from other Intensive sessions

In the first step, our results of the Southern Intensives (SI vie) are investigated in comparison with the in-house solution for the northern hemisphere IVS Intensives INT1 and INT3 (INT1/3 vie) and for Russian Intensives (RI vie). Thereby, we can ensure consistency and avoid biases, possibly arising just from different settings in the session analysis. As mentioned in section “Scheduling and simulations”, most of the SI were observed simultaneous with an INT1 session (at 18:30 UTC), for these we assume the reference epoch to be identical. Since the effective mid of the sessions does not necessarily correspond to the nominal middle, this is not completely true, but the variation of UT1 within these subtle time intervals amounts to 2.5 µs in single cases and mostly ranges below 0.5 µs. We considered the size of the values to be acceptable. The same issue with reference epochs being slightly shifted applies when comparing the SI results with external INT1 solutions or the different INT1/3 results among each other. Other AC specify the reference epoch with respect to the nominal session start and the actual session duration, while the vie solution takes the middle between the first and last scan as used in the analysis. The impact of the small epoch differences on UT1−UTC is at an equal scale to or smaller than for the SI versus INT1 vie epochs.

For comparing with the SI starting at 15:30 UTC, the UT1−UTC estimates of the surrounding INT1 and INT3 sessions are interpolated linearly to the SI epochs. When examining intrinsic properties of INT1/3 solutions, such as mean formal errors, we avoided interpolation and used the original samples, accepting at the same time that the reference epochs of the sessions with the same nominal session start are not fully congruent, as explained above.

**Fig. 5:**
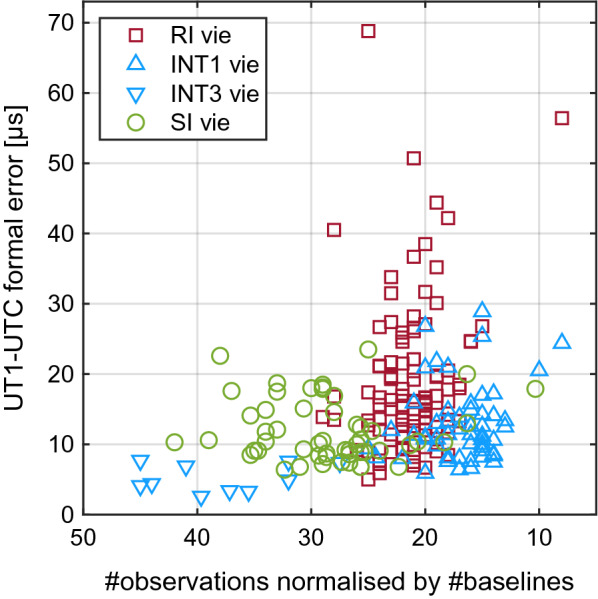
Number of observations per session normalised by number of baselines versus UT1−UTC formal error [µs]

Before looking at the UT1−UTC values, we will explore the session performance of the SI in terms of number of observations and the formal errors of the UT1−UTC results. In Fig. 5, the numbers of observations per baseline are plotted against the UT1−UTC formal errors per session for each of the three Intensive types SI, INT1/3 and RI. It should be noted here, that 17 out of 53 sessions were observed or analysed only on one baseline, mostly the HtYg baseline, because of teething problems with the VGOS equipment at Hobart, which were mostly resolved in 2021. However, this discrepancy and the fact that most of the INT3 are observed with more than two stations should be extensively balanced by normalising the observation numbers by the number of baselines. The horizontal axis showing the observation numbers is flipped from left to right, because we would expect a lower number of observations to correlate with a higher formal error.

Compared to INT1, we see a much higher number of observations used in the SI analysis, mostly explained by the 1 Gbps observing mode used for SI, as compared to the 128 Mbps observing mode used for INT1 as well as the faster slewing telescopes of the SI network. Despite the INT1 managing with less observations, their formal errors are in the same range as those of the SI sessions, a fact that could be explained by the exceptional advantageous geometry of the INT1 baselines. Based on the studies on baseline geometry by Schartner et al. (2021a), we can infer that the mean formal error of the Wettzell–Kokee baseline is about half of the mean formal error expected for the less advantageous SI baseline HtYg, and around two-thirds of that for HtHb. Most likely the geometrical disadvantages of the SI and shortcomings of the ICRF in the southern hemisphere are compensated by the larger number of observations. The mean and median values of the observation numbers and of the UT1−UTC formal errors for the three vie solutions are given in Table 1. As can be seen in the table as well as from Fig. 5, the RI have slightly more observations than the INT1 but still less than the SI sessions. Although the WRMS of the post-fit residuals and the formal errors of the observations of the RI are much lower than for SI or INT1/3 (see Fig. 3 and Table 1), the UT1−UTC uncertainties are generally higher. From a pure geometrical point of view, the RI baselines have the most disadvantageous preconditions. The figures in the work of Schartner et al. (2021a) report that the simulated mean formal error of the most employed Russian baseline Badary–Zelenchukskaya is almost three times larger than that of the optimal INT1 baseline Kokee–Wettzell. In contrast to the SI sessions, the geometrical handicap of the RI is not adjusted with a higher number of observations.

**Table 1:** Mean and median values of the number of observations normalised by the number of baselines (#obs), of the formal errors of the observations ($\sigma _{\mathrm {obs}}$) and of the formal errors of the UT1−UTC estimates ($\sigma _{\mathrm {UT1}}$)

Series	Mean	Median	Mean	Median	Mean	Median
	#obs	#obs	$\sigma _{\mathrm {obs}}$ [ps]	$\sigma _{\mathrm {obs}}$ [ps]	$\sigma _{\mathrm {UT1}}$ [µs]	$\sigma _{\mathrm {UT1}}$ [µs]
SI scheduled	35	34	–	–	–	–
SI simulated	35	34	–	–	10	10
SI vie	28	29	36	32	12	10
INT1/3 vie	20	18	36	30	12	11
RI vie	21	21	14	11	19	16

**Fig. 6:**
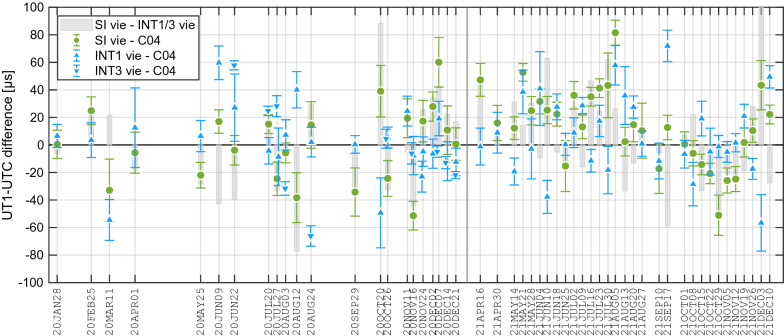
Differences between SI vie and INT1/3 vie UT1−UTC results and with respect to IERS 14C04 (C04) in µs. The differences SI vie minus INT1/3 vie are printed as grey bars. The error bars show the formal errors of the Intensive sessions. Green circles illustrate SI vie minus C04 results, blue upward/downward pointing triangles INT1/3 vie minus C04 values. The labels on the horizontal axis are the dates of the SI

In Fig. 6, the time series of estimated UT1−UTC from the solutions SI vie and INT1/3 vie, including their formal uncertainties as error bars, are printed w.r.t. the C04 values. The Intensive internal differences, SI vie minus INT1/3 vie, are displayed as bars in the background. The range of deviations to the C04 of the SI vie series is comparable to the INT1/3 vie differences to C04, with a WRMS scatter amounting to about 29 µs for both Intensive types. Virtually the same scatter results for SI vie minus INT1/3 vie, where we obtain a WRMS of 31 µs.

**Fig. 7:**
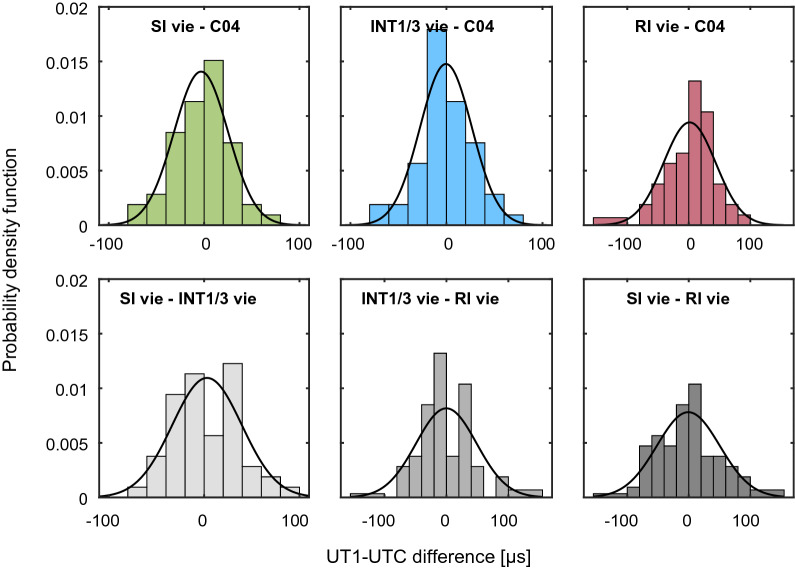
Histograms of UT1−UTC differences [µs] with fitted normal distribution (solid black lines), normalised as probability density function

The unbiased spread of the differences SI vie, INT1/3 vie and RI vie versus C04 and of the differences among the Intensives is illustrated in Fig. 7 with six histograms in terms of the probability density function. For these comparisons the INT1/3 vie and RI vie series are interpolated to the SI epochs. All differences are bias-corrected by removing a weighted mean before calculating the probability density functions. The series differences are all reasonably close to normal distributions, as can be deduced by comparing with the black curves, which represent normal distribution fitted to the data. The histograms show the deviations in steps of 20 µs, with the largest difference amounting to 100 µs for the SI versus INT1/3 case. Whenever the RI are involved the discrepancies are larger, partly exceeding 150 µs. Within the Intensive internal comparisons the distribution of the differences is wider, which demonstrates that the average discrepancies between the Intensive series internally are larger than between the single Intensive series and C04.

In the following the examination of the UT1−UTC output of the Southern Intensives is extended by comparison with the INT1/3 results of other AC. Different solutions of the same Intensives INT1/3 are investigated to get an estimate of the effect of diverging analysis settings on the UT1−UTC results, which becomes visible in the agreement of the AC solutions. We selected INT1/3 solutions from four external AC, namely from the Federal Agency for Cartography and Geodesy in Germany (bkg), NASA Goddard Space Flight Center (gsf), Paris Observatory (opa) and the United States Naval Observatory (usn). The choice of AC was made depending on the complete availability of the UT1−UTC values for the same 64 sessions as included in the INT1/3 vie solution. The differences between UT1−UTC values are assessed by means of weighted mean (WMEAN) and weighted standard deviation (WSTD), which are also built w.r.t. the C04 series. The precision of the different series is compared via the mean formal errors (MFE). Additionally, we employed another alternative indicator for the accuracy of the UT1−UTC series, calculated with the so-called three-cornered hat method (3CH). This method was primarily developed by Gray and Allan (1974) to investigate the random errors of atomic clocks. In its simplest form it can be used for the estimation of the error variances of three independent time series supposed to measure the same physical quantity. In our case, we only dispose of three networks measuring UT1−UTC independently, albeit minute correlations might be generated when there is an overlap of the radio sources observed. We suppose that the variances of the three independent data sets SI, INT1/3 (=INT), and RI are related in the following way:

4 $$\begin{aligned} \begin{aligned} \sigma ^{2}_{\mathrm {SI-INT}}&=\sigma ^2_\mathrm {SI}+\sigma ^2_\mathrm {INT}\;,\\ \sigma ^{2}_\mathrm {SI-RI}&=\sigma ^2_\mathrm {SI}+\sigma ^2_\mathrm {RI}\;,\\ \sigma ^{2}_\mathrm {INT-RI}&=\sigma ^2_\mathrm {INT}+\sigma ^2_\mathrm {RI}\;. \end{aligned} \end{aligned}$$

Consequently, we can derive the single variances by recombination:

5 $$\begin{aligned} \begin{aligned} \sigma ^{2}_{\mathrm {SI}}&=(\sigma ^2_\mathrm {SI-INT}+\sigma ^2_\mathrm {SI-RI}-\sigma ^2_\mathrm {INT-RI})/2\;,\\ \sigma ^{2}_{\mathrm {INT}}&=(\sigma ^2_\mathrm {SI-INT}+\sigma ^2_\mathrm {INT-RI}-\sigma ^2_\mathrm {SI-RI})/2\;,\\ \sigma ^{2}_{\mathrm {RI}}&=(\sigma ^2_\mathrm {SI-RI}+\sigma ^2_\mathrm {INT-RI}-\sigma ^2_\mathrm {SI-INT})/2\;.\\ \end{aligned} \end{aligned}$$

The square roots of the variances (hereafter designated 3CH) reflect the accuracy of the individual series, independently of the respective adjustment model used in the estimation process. The index INT stands for any of the INT1/3 solutions of the five AC (vie, bkg, gsf, opa, usn). Equation (5) can therefore be evaluated five times for SI and RI. Hence the 3CH figures for SI and RI are calculated as mean values from the evaluations with the five AC difference variances. A multi-solution 3CH analysis including all INT solutions would fail (by yielding negative variances) due to the high correlations, stemming from the identical observation setup.

**Fig. 8:**
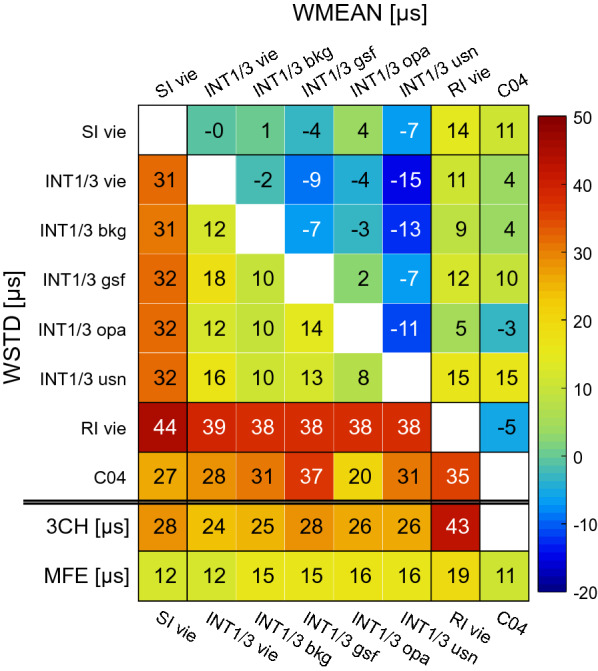
Heatmap of comparison parameters for different UT1−UTC time series. Right triangular matrix: weighted means (WMEAN) of UT1−UTC time series differences, sign convention is row minus column. Left triangular matrix: weighted standard deviations (WSTD) of UT1−UTC time series differences. Bottom: UT1−UTC accuracy from three-cornered hat analysis (3CH) and UT1−UTC mean formal errors (MFE) of the different time series corresponding to the column labels. The colours visualise the range in addition to the values printed in the individual fields. All numbers are given in µs

The numbers for all comparison measures mentioned before (WMEAN, WSTD, 3CH, MFE) are compactly presented as a kind of heatmap in Fig. 8. The biases, expressed as WMEAN, of the SI to the different INT1/3 solutions are rather small, with an absolute maximum of 7 µs. Biases between the different AC solutions show larger numbers, up to 15 µs in absolute values. The WMEAN of SI minus C04 is 11 µs, which is more than SI−INT1/3, but well in the range of the WMEAN of the other Intensive solutions w.r.t. C04. Presumably, this offset is a reference frame effect because the positions of the Australian stations we use in the vievsTrf differ notably from the positions in ITRF2014, where the C04 series is aligned to. The biases with the RI are of similar size as the biases between the different AC solutions or the biases with C04.

As to the WSTD, the situation is rather opposite to the WMEAN picture. We find consistent agreement of the different AC for the INT1/3 sessions in the range of 8–18 µs, but clearly higher WSTD w.r.t. the SI, where the values smoothly average to 32 µs. Ultimately, these results are not surprising, since all AC process the same observations to derive UT1−UTC, while SI results are obtained from completely independent observations. The WSTD of the SI w.r.t. the benchmark series C04 is again smaller, coming up to 27 µs, whereas this WSTD level is around 30 µs for the INT1/3 sessions, with positive and negative excursions of gsf (37 µs) and opa (20 µs), respectively. In terms of WSTD the RI exhibit the largest values with the maximum of 44 µs found w.r.t. the SI. The deviation w.r.t. the INT1/3 solutions averages to 38 µs. One, yet very small, portion of the high WSTD values can be attributed to the error that is made when interpolating linearly from the RI to the SI epochs. The higher noise level of the RI is also confirmed through a higher mean formal error.

As explained in the previous paragraph, the RI UT1−UTC results are used to estimate a kind of alternative standard deviation, the 3CH. The 3CH value of the RI is between the WSTD of the differences to the INT1/3 and the SI time series. For the different INT1/3 solutions, we find a variation of the 3CH values from 24 µs to 28 µs and a value of 28 µs for the SI. The level of uncertainty indicated by the 3CH analysis generally shows better agreement with the WSTD scatter of the series differences to C04 than with their mean formal errors, which are 12 µs for the SI, between 12 µs and 16 µs for INT1/3 and 19 µs for the RI. This is another evidence for the UT1−UTC formal errors being too optimistic, as it is commonly presumed.

### Intrinsic assessment of SI single baselines

**Fig. 9:**
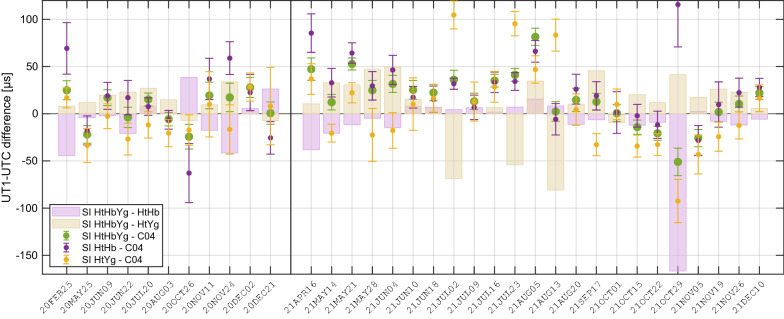
Comparison of UT1−UTC derived from the SI full network HtHbYg and the SI single baselines HtHb and HtYg and with respect to C04 in units of µs. Dots with error bars illustrate the differences to C04, with the error bars reflecting the UT1−UTC formal errors of the corresponding series: HtHbYg (green), HtHb (purple), HtYg (yellow). Purple and yellow bars show the UT1−UTC differences of the full network solution HtHbYg and the single baseline solutions HtHb and HtYg

In consideration of the very limited data transfer velocity from Yg to Hb (see section “Observation and correlation”), we have to accept that an Intensive session involving the baseline HtYg will not provide UT1−UTC results within hours after the observation. This implies in turn, that a routine Southern Intensive session with quick turnaround times can only be established on the HtHb baseline. Thus we have to ascertain that the overall accuracy and performance is equally backed by the single HtHb baseline and not, for instance, dominated by the HtYg baseline. To go into that matter, we extract a subset of the SI sessions, picking only dates when all three stations were observing. This requirement applies for 11 sessions in 2020 and for 23 in 2021. The 34 sessions are additionally analysed separately using only the observations of the HtHb and the HtYg baseline. The UT1−UTC values resulting from the single baselines are compared with the solution of the full three station constellation HtHbYg as well as with the C04 series. The individual calculated UT1−UTC differences for each of the selected sessions are plotted in Fig. 9. The distribution of the differences in Fig. 9 already indicates that the three station solution is primarily determined by the HtHb baseline (disregarding the outlier on October 29 in 2021). In terms of WSTD (HtHbYg-HtHb: 16 µs, HtHbYg-HtYg: 29 µs), we see a closer alignment of the HtHb baseline results to the full network results. The dominating role of the baseline with Hobart is also confirmed by the WSTD of all SI versions’ differences to C04, which are 25 µs for HtHbYg and HtHb and 39 µs for HtYg. The bias w.r.t. C04, expressed as weighted mean, is however least for HtYg with 4 µs, in contrast to 15 µs and 23 µs for HtHbYg and HtHb, respectively. In summary, the HtHb baseline shows better agreement than the HtYg baseline, both with the triple baseline solution and with the C04 UT1−UTC series. The apparently superior performance of the HtHb baseline is further supported by precision measures. Although the median number of observations is only 24 for HtHb versus 30 for HtYg, the respective mean formal errors are 16 µs and 18 µs. The generally worse performance of the HtYg baseline, can be somewhat explained by the HtHb baseline featuring a superior geometry for UT1−UTC determination (Schartner et al. 2021a).

## Conclusions and future perspectives

The VLBI Intensive sessions are undoubtedly the best option to estimate and provide the parameter UT1−UTC with acceptable latency and accuracy. One drawback of the nowadays routinely performed Intensive sessions, however, is the geographic bias in the global distribution of the involved telescopes. The stations observing either traditional Intensives or VGOS Intensives are all located north of the equator. The mitigation of systematic effects potentially caused by this asymmetry and the generation of an independent verification possibility for established Intensive results motivated the constitution of the Southern Intensives program. We present the results of 53 out of more than 60 SI sessions, which have been observed since January 2020, with observations continuing in 2022. The SI sessions are operated using three antennas, Ht in South Africa and Hb and Yg belonging to the AuScope VLBI array in Australia. As a kind of byproduct, though essential for UT1−UTC consistency, the so-called peculiar offset was successfully determined for the new receiving system in Hobart within the correlation process of seven SI sessions in 2020.

The quality and the performance of the SI are rated by comparison with results of INT1 and INT3 sessions of five different analysis centers, results of the Russian Intensives, and the IERS EOP 14C04 series. The WSTD agreement of SI vie with C04 and with the INT1/3 solutions of different AC is $\sim$ 30 µs. This is at the same level as the WSTD of the differences INT1/3 minus C04, ranging between 20 µs and 37 µs for the different AC. The differences of SI vie minus RI vie have a higher WSTD, but this is also the case for the WSTD values of the RI differences with INT1/3 or C04. The higher scatter of the RI and the larger UT1−UTC formal errors suggest that the precision of these sessions is lower. Since the RI are observed on the shortest baselines, among the Intensive types compared here, a weaker UT1 sensitivity can be expected. The inspection of the histograms of the differences SI vie minus C04, SI vie minus INT1/3 vie and SI vie minus RI vie does not indicate any systematics, and the bias-free UT1−UTC differences clearly follow a normal distribution.

The biases (WMEAN) of the SI series w.r.t. the INT1/3 series of the five AC are rather small and the fact that they exhibit different signs suggests that we do not see any systematic North-South bias for the observed time period. Comparing with C04 or RI, the SI show WMEAN values above 10 µs, which are however not extraordinary, considering WMEAN of up to 15 µs in absolute numbers obtained when comparing different INT1/3 solutions among each other and w.r.t. C04. We believe that the main part of the observed biases among the Intensive series and with C04 can be attributed to inconsistent underlying terrestrial reference frames. The EOP 14C04 are aligned to ITRF2014, while that frame is used by the VLBI AC only to a certain extent, since there are also stations participating in the IVS INT sessions which do not have ITRF2014 coordinates. For INT1/3 vie and SI vie processing we employed a VieVS internal terrestrial reference frame, based on longer time series than ITRF2014, which is especially important for the Australian telescopes. The RI vie series are derived with the same TRF solution as SI vie and INT1/3 vie. Therefore we can not justify larger biases between all vie solutions with inconsistent station coordinates. For a thorough investigation of the significance and origin of the biases with the RI a longer study period would be appropriate.

As an alternative indicator of the UT1−UTC accuracy we consult the 3CH values derived by means of a three-cornered hat analysis of the Intensive time series. The 3CH numbers are approximately at the level of the WSTD of the different Intensive types and solutions w.r.t. each other as well as of all series differences to C04. The standard deviations of UT1−UTC resulting from the adjustment procedures are summed up here as mean formal errors. The MFE are ranging from 12 µs (SI vie, INT1/3 vie) up to 19 µs (RI vie). The formal errors of UT1−UTC are commonly deemed too optimistic. Seeing that the gross of WSTD scatter and also the 3CH scores roughly correspond to the twofold MFE of the UT1−UTC series, this assumption turns out to be reasonable.

Based on all the discussed quality aspects, we conclude that the Southern Intensives definitely stand comparison with the established IVS Intensives in the northern hemisphere. Taking into account that technical difficulties, leading to a 18 % loss of SI observations, could be resolved during the year 2021, we see the potential for further improvement of the SI standard deviation. Further prospects for a gain in precision rely on the augmentation of the celestial reference frame in the South. In a quick-look survey, we repeated Intensive baseline simulations as presented by Schartner et al. (2021a, Fig. 2) for stations in the northern hemisphere, for baselines from a reference station of 30° southern latitude (representative for Ht) to all possible second stations on a 10° by 10° grid (0°–180° longitude, −80° to 80° latitude). Subsequently we can compare the theoretical standard deviations for Intensive baselines, extending from 30° North to 30°/40° North over a longitudinal distance of 90°/130°, to the same configuration in the South, mirrored at the equator, which corresponds to the SI baseline geometry of HtYg and HtHb, respectively. From the difference map we can infer an approximate improvement of the standard deviation of up to 16 % for the HtYg baseline and up to 20 % for the HtHb baseline, if the CRF in the South would catch up to the quality of that in the North.

Another important outcome of this study is that a Southern Intensive session observed on the HtHb baseline only is equally able to compete with existing INT sessions. A comparative analysis of a SI session subset gives strong evidence for the HtHb baseline dominating the three station solution. The WSTD with C04 does not change when UT1−UTC is estimated from HtHb instead of HtHbYg, whereas for HtYg it increases by 14 µs. This supremacy of the baseline with Hobart is in fact beneficial for the SI program in view of a routine operation that should provide rapid service UT1−UTC. The general concept of e-VLBI and ultra-rapid operations for Earth rotation determination is described in Sekido et al. (2008) and Haas et al. (2017). Since the station Yarragadee does not facilitate e-transfer of the observation data, UT1−UTC cannot be delivered rapidly from the full SI baseline configuration. At present or more precisely for the SI sessions in 2022 this issue is resolved insofar as the HtHb baseline data are correlated and provided right after the observation, while the Yg data are added afterwards, as soon as they arrive at the correlator. The 2022 SI are scheduled every Monday at 6:30 UTC, which is overlapping with the INT3 starting at 7:00 UTC. The sessions are listed in the IVS Intensive master schedule^3^ and the corresponding files are provided to the international community via the Crustal Dynamics Data Information System (CDDIS, Noll 2010). Current emphasis of the SI program is to improve automation and routinely deliver reliable data with low latency, before hopefully in the future this program can be expanded to a higher cadence.

## Data Availability

SI session master files: https://www.vlbi.at/data/scheduling/southern_intensives/ SI vgosDB files: https://www.vlbi.at/data/correlation/public/vgosdb/ SI, INT1/3 and RI results of AC vie and solution description: https://www.vlbi.at/data/analysis/southern_intensives/ RI databases were downloaded from: ftp://quasar.ipa.nw.ru/pub/EOS/IAA/ngs/ INT results of the IVS AC: https://cddis.nasa.gov/archive/vlbi/ivsproducts/eopi/.

## Abbreviations

AC: Analysis center
GPS: Global Positioning System
EOP: Earth orientation parameters
HartRAO: Hartebeesthoek Radio Astronomy Observatory
ICRF: International Celestial Reference Frame
IERS: International Earth Rotation and Reference Systems Service
ITRF: International Terrestrial Reference Frame
IVS: International VLBI Service for Geodesy and Astrometry
MFE: Mean formal error
NNR: No net rotation
NNT: No net translation
RI: Russian Intensive
SI: Southern Intensive
3CH: Three-cornered hat
USNO: United States Naval Observatory
UT1: Universal Time 1
UTC: Coordinated Universal Time
VGOS: VLBI Global Observing System
VieVS: Vienna VLBI and Satellite Software
VLBI: Very Long Baseline Interferometry
WMEAN: Weighted mean
WRMS: Weighted root mean square
WSTD: Weighted standard deviation

## Footnotes

1 https://github.com/whi-llc/adjust.

2 https://vlbi.at/data/analysis/ggrf/trf_vie2020_211030_withVGOS.TXT.

3 https://ivscc.gsfc.nasa.gov/sessions/intensive/2022/.
